# Bone Health in Patients With Type 2 Diabetes

**DOI:** 10.1210/jendso/bvae112

**Published:** 2024-06-06

**Authors:** Patrice Forner, Angela Sheu

**Affiliations:** Clinical School, Faculty of Medicine, St Vincent's Hospital, University of New South Wales Sydney, Sydney, NSW 2010, Australia; Department of Endocrinology and Diabetes, St Vincent's Hospital, Sydney, NSW 2010, Australia; Clinical School, Faculty of Medicine, St Vincent's Hospital, University of New South Wales Sydney, Sydney, NSW 2010, Australia; Department of Endocrinology and Diabetes, St Vincent's Hospital, Sydney, NSW 2010, Australia; Skeletal Diseases Program, Garvan Institute of Medical Research, Darlinghurst, NSW 2035, Australia

**Keywords:** diabetes, fractures, osteoporosis, bone, insulin resistance

## Abstract

The association between type 2 diabetes mellitus (T2DM) and skeletal fragility is complex, with effects on bone at the cellular, molecular, and biomechanical levels. As a result, people with T2DM, compared to those without, are at increased risk of fracture, despite often having preserved bone mineral density (BMD) on dual-energy x-ray absorptiometry (DXA). Maladaptive skeletal loading and changes in bone architecture (particularly cortical porosity and low cortical volumes, the hallmark of diabetic osteopathy) are not apparent on routine DXA. Alternative imaging modalities, including quantitative computed tomography and trabecular bone score, allow for noninvasive visualization of cortical and trabecular compartments and may be useful in identifying those at risk for fractures.

Current fracture risk calculators underestimate fracture risk in T2DM, partly due to their reliance on BMD. As a result, individuals with T2DM, who are at high risk of fracture, may be overlooked for commencement of osteoporosis therapy. Rather, management of skeletal health in T2DM should include consideration of treatment initiation at lower BMD thresholds, the use of adjusted fracture risk calculators, and consideration of metabolic and nonskeletal risk factors. Antidiabetic medications have differing effects on the skeleton and treatment choice should consider the bone impacts in those at risk for fracture.

T2DM poses a unique challenge when it comes to assessing bone health and fracture risk. This article discusses the clinical burden and presentation of skeletal disease in T2DM. Two clinical cases are presented to illustrate a clinical approach in assessing and managing fracture risk in these patients.

There is growing evidence that type 2 diabetes mellitus (T2DM) is associated with poor bone health. Increased body weight has been regarded as protective against osteoporosis as a result of increased mechanical loading, particularly at weight bearing sites. However, despite a general trend toward higher body mass index (BMI), patients with T2DM tend to have overall poor bone health, with an increased risk of some fractures and, importantly, worse postfracture outcomes, including delayed fracture healing and increased mortality [1-3]. The underlying contributors are complex but include maladaptive skeletal loading and reduced resistance to stress loading, as a result of altered bone microarchitecture and low bone turnover, due to direct and indirect effects of hyperglycemia, obesity, accumulation of advanced glycation end products (AGEs), and vascular disease. This review will summarize the clinical and pathophysiological features of diabetic osteopathy and uses 2 cases to illustrate clinical assessment and management of these patients.

## The Clinical Burden of Skeletal Fragility in T2DM

### Risk of Fractures in T2DM

A meta-analysis of fracture risk in T2DM showed an increased risk of any fracture (risk ratio [RR] 1.2) [4, 5], particularly of the hip (RR 1.3 - 2.1) [4-7]. This association remained after controlling for age, physical activity, and BMI. There are limited studies on non-hip, nonvertebral fractures in T2DM, with inconsistent findings, although there was an increased risk of wrist [5] and foot fracture [4] in 2 separate meta-analyses.

The effect of T2DM on vertebral fractures remains unclear. Early meta-analyses showed no increased risk in T2DM, although these studies have several limitations, including differences in ascertainment of fractures [4, 5, 7]. A recent meta-analysis of 15 studies showed that individuals with T2DM had a lower risk of prevalent (odds ratio [OR] 0.84 [95% CI, 0.74-0.95]) but increased risk of incident vertebral fractures (OR 1.35 [95% CI, 1.27-1.44]) [8]. Since people with T2DM may have radiographs more frequently than those without T2DM, incident vertebral fractures may have been more frequently detected in study participants with T2DM. Importantly, the presence of any vertebral fracture was associated with increased risk of nonvertebral fractures and increased mortality, confirming the importance of vertebral fractures and suggesting that routine vertebral radiography may be useful in people with T2DM. However, there was significant heterogeneity in studies, due to differences in fracture ascertainment, including self-report, clinical radiography, and systematic routine radiography. Despite some studies showing no difference in vertebral fracture risk in T2DM compared to those without [4, 5, 7], the meta-analysis found an overall elevated risk even when adjusted for heterogeneity (OR 1.55 [95% CI, 1.04-2.31]). Whether vertebral fractures are more common in people with T2DM, or if this reflects more frequent clinical radiography in people with T2DM, remains unclear, and a prospective study with routine radiographs would be enlightening.

### T2DM-Related Clinical Risk Factors Associated With Fracture

One of the challenges of interpreting fracture risk in T2DM is the lack of dedicated prospective studies examining fracture outcomes in cohorts adequately powered for fracture with concurrent detailed metabolic characterization. Epidemiological studies suggest that elevated fracture risk is limited to a subgroup of individuals with T2DM, particularly those with microvascular complications, on insulin therapy, and with poorer glycemic control [9-11]. However, the data has been conflicting and the independent associations of these often-coexisting factors are difficult to determine in the absence of fully adjusted modeling.

#### Duration of diabetes

Longer duration of T2DM is mostly associated with increased fracture risk [9, 12], with one study finding decreased fracture risk in patients with newly diagnosed T2DM compared to controls without T2DM [13]. This is in keeping with a reduction in fracture risk in those with prediabetes, potentially owing to improved bone mineral density (BMD) prior to established and prolonged hyperglycemia [14], although the definition of prediabetes is variable across studies and may explain discrepant findings [15].

#### Glycemic control

The relationship between glycemic control and fracture risk also remains an area of contention. While hyperglycemia has been associated with an increased risk of fracture, these findings are confounded by a number of variables, including the use of antidiabetic medications, particularly insulin therapy, and presence of diabetes-related complications [9, 11]. An increased risk of hip fracture has been described in older subjects with tight glycemic control (glycated hemoglobin [HbA1c] < 7.0%), compared to those with an HbA1c of > 8%, possibly owing to hypoglycemia-related falls [16]. In contrast, a retrospective cohort study of patients with T2DM aged 65 years and over showed an increasing trend between HbA1c and hip fractures with a 24% to 31% higher risk of hip fracture in subjects with a HbA1c of ≥ 9% compared to those with an HbA1c of 6% to 7% [17]. Similarly, an increased risk of fracture was noted at extremes of HbA1c with both levels < 6.5% and > 9.5% associated with an increased risk of fracture [18]. Postulated mechanisms include cumulative hyperglycemia (for elevated HbA1c) and hypoglycemia-related falls (for lower HbA1c). Additionally, the impact of day-to-day variability HbA1c at the time of the fracture (rather than at study entry) is currently unknown.

#### Microvascular complications

Increased fracture risk has been strongly associated with microvascular complications [9, 19]. Mechanisms have been linked to poor bone microarchitecture and also indirectly due to increased risk of falls associated with peripheral neuropathy and diabetic retinopathy. Many studies of microvascular complications and fracture risk are confounded by a typically longer disease duration and suboptimal glycemic control that predisposes individuals to microvascular complications in the first place. Nevertheless, increased fractures have been independently associated with microvascular complications, confirming the importance of vascular disease in bone health [19]. In a post hoc analysis of the Fenofibrate Intervention and Event Lowering in Diabetes (FIELD) study, insulin and vascular complications (macrovascular disease in men and microvascular disease in women) were independently associated with fragility fractures, even when baseline glycemic control and T2DM duration were accounted for [19]. Since bone is a highly vascular organ and there is evidence of bone changes associated with vascular complications (see “High-resolution peripheral quantitative computed tomography” in the section “Measurement of Bone Parameters in Type 2 Diabetes” below), it is highly likely that vascular complications are directly associated with impaired bone (see section “Pathophysiology of Skeletal Fragility in T2DM” below). Whether there are additional contributions from nonbone factors such as falls has not yet been excluded.

#### Insulin therapy

Insulin therapy has been consistently shown to be associated with increased risk of fractures [9, 20, 21]. As with glycemic control and microvascular complications, insulin therapy is generally reserved for patients with more complicated T2DM, and therefore its independent association with fracture risk is particularly difficult to determine in the absence of a prospective randomized controlled trial (RCT). Additionally, since insulin is anabolic to bone, there is a question as to whether there are indirect factors such as hypoglycemia-related falls that may drive fractures. The results from both the FIELD study [19], which showed independent associations of insulin therapy with any fractures and particularly distal fractures, and the evidence for a J-curve association with HbA1c [17], both support falls as a potential driver for fractures due to insulin therapy. Similarly, a Swedish national cohort study investigating fracture risk in T2DM showed a marginally but significantly increased risk of major osteoporotic fracture (HR 1.01 [95% CI, 1.00 to 1.03]) compared with controls [12]. However, the following 4 risk factors for substantially higher risk of fracture (20%) were identified: low BMI, long duration of T2DM, insulin treatment, and low physical activity. Since the majority of the subjects had none of these risk factors, the authors postulated this as the cause for the low risk of fractures overall.

Together, these data suggest that skeletal fragility in T2DM is heterogenous, with not all people with T2DM having the same risk for fracture, and this may explain the differences observed between the clinically diverse cohorts examined in studies. It highlights the importance of adequately controlling for multiple clinical and metabolic characteristics to better define the skeletal phenotype of T2DM.

### Postfracture Outcomes in T2DM

Individuals with T2DM experience worse postfracture outcomes. There is often an increased risk of infection, delayed wound healing, and diminished return to independent functionality in those with T2DM [22]. In the general population, fractures are associated with increased mortality [23, 24]. Concerningly, postfracture mortality is further increased in T2DM (30% to 50%) following any fracture, with a cumulative effect on mortality that is higher compared with either T2DM or fracture alone [3]. Importantly, while hip and vertebral fractures are associated with the greatest mortality burden, non-hip, nonvertebral fractures are also associated with increased mortality, and thus every fragility fracture should be regarded as a sentinel event for targeted management. Since non-hip, nonvertebral fractures are the most common sites for fracture, the clinical burden of diabetic osteopathy is high, and treatment should be considered following all fragility fractures in people with T2DM.

## Pathophysiology of Skeletal Fragility in T2DM

The underlying metabolic contributors to skeletal fragility are complex [25]. The potential benefits of skeletal loading from higher BMI, which often coexists in people with T2DM, is offset by a number of T2DM-related factors including hyperglycemia, hyperinsulinemia, AGEs, and chronic proinflammatory state [26]. Overall, these metabolic factors suppress bone formation and impair bone microarchitecture, resulting in decreased strength loading and increased susceptibility to fracture despite relatively preserved BMD. Additionally, nonskeletal factors also contribute to fracture risk; although obesity may be protective for hip fractures, which has been postulated to be due to the shock absorbing cushioning effects of increased hip adiposity, there is a higher risk of distal limb fractures in obese patients, likely due to greater forces from higher body weight being transmitted during a fall [27].

### Obesity

Obesity is a proinflammatory state. Chronic inflammation has a direct effect on the bone microenvironment with interleukin-6 and tumor necrosis factor alpha activating the RANK pathway and stimulating osteoclasts resulting in increased bone resorption [28, 29]. Additionally, there are complex, still unclarified, data on the effect of adipokines on bone health, particularly in the context of fluctuating weight and metabolic health. Obesity is associated with high levels of the proinflammatory adipokine leptin and low levels of the anti-inflammatory adipokine adiponectin, while the reverse (lower leptin and higher adiponectin) is seen in caloric restriction and starvation [30]. Two meta-analyses showed a positive association between leptin, BMD, and incident fracture [31, 32]. Adiponectin levels have been negatively associated with BMD, although with discordant fracture risk [31, 32]. Together, the balance of increased skeletal loading, offset by chronic inflammation, higher levels of leptin, and lower levels of adiponectin, may have negative bone effects.

### Hyperglycemia

Hyperglycemia directly affects the bone microenvironment by suppressing osteoblast maturation and differentiation, resulting in demineralization of trabecular bone and reduced bone formation [33, 34]. Hyperglycemia also impairs mechanosensing leading to impaired skeletal strength through acceleration of osteocyte senescence and apoptosis [33, 34]. Together, hyperglycemia negatively affects bone cells, leading to poorer bone formation and strength resistance.

### Advanced Glycation End Products

AGEs have been associated with the pathogenesis of diabetes-related complications, including micro- and macrovascular disease. Levels of AGEs are elevated in T2DM as a result of chronic hyperglycemia and oxidative stress. There is increasing evidence that AGEs play a significant role in bone fragility by impairing bone mineralization, through disruption of cross-linkage of collagen and proteins, and also directly through the inhibition of osteoblast proliferation and the induction of osteoblast apoptosis, with effects on both cortical and trabecular bone [35]. Activation of the AGE receptor results in the production of reactive oxygen species and inflammatory cytokines, resulting in chronic inflammation and bone resorption [36]. Clinically, assessment of AGEs in vivo is challenging, although preclinical data suggest that AGEs have a negative effect on bone microarchitecture, bone density, and bone strength [35, 37]. AGEs may be the link between the development of vascular complications and increased fractures in T2DM. Whether reducing AGEs improves bone parameters and reduces fracture risk remains to be elucidated.

## Case 1: Assessing Skeletal Fragility in T2DM

**Figure 1.**
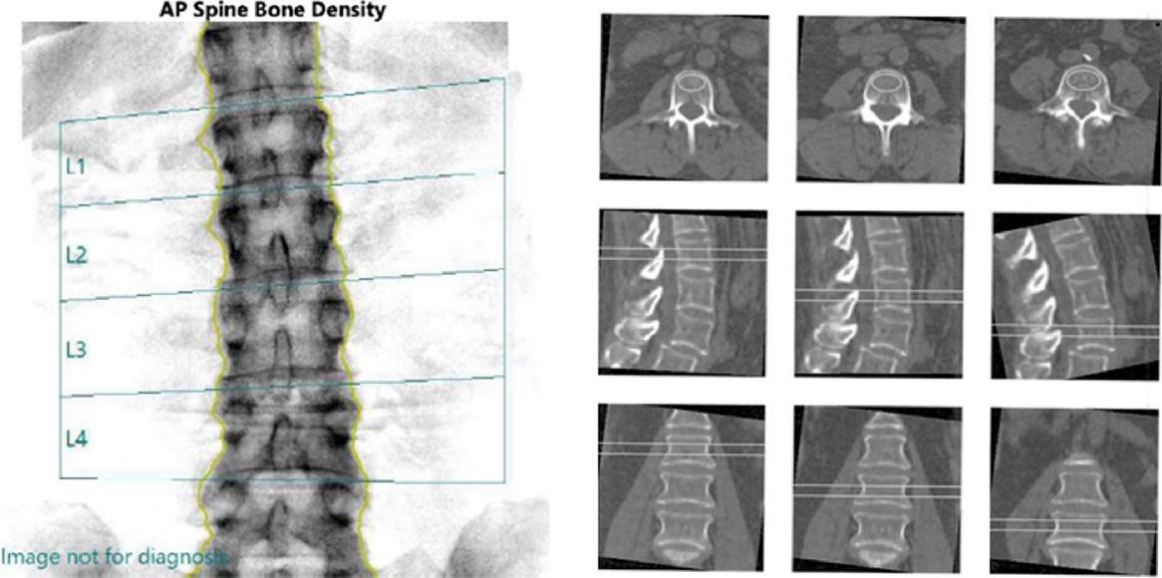
Case 1, lumbar spine BMD using DXA and QCT.

A 59-year-old woman was reviewed for management of osteoporosis. Her comorbidities included T2DM, hypertension, hypercholesterolemia and obesity (BMI of 32 kg/m^2^). She had sustained a fracture of her right radius following a fall from standing height and a coccygeal fracture after a fall down one stairstep. Following this, she commenced risedronate 35 mg weekly. After 3 years of treatment, she was investigated for atraumatic right ankle pain. Imaging revealed a right medial malleoli fracture, requiring surgical fixation. She was replete with 25(OH)vitamin D and there were no secondary abnormalities detected (calcium/PTH/TSH within reference intervals, normal serum electrophoresis). Her HbA1c was 8.6%. Her BMD on DXA corresponded to a T-score of −1.0 at the lumbar spine (L2-L4) and T-score of −0.3 at the left total hip (see Fig. 1). The trabecular bone score (TBS) was 1.20, consistent with significant microarchitecture degradation. The spine quantitative computed tomography (QCT) scan revealed a T-score of −3.1 over L2-L4. Table 1 summarizes the QCT and DXA results at the lumbar spine. In light of her fracture on risedronate she was commenced on romosozumab.

**Table 1.** Case 1 lumbar spine BMD results using DXA and QCT

ROI	DXA		QCT	
	Areal BMD (g/cm^2^)	T-score	Volumetric BMD (mg/cm^3^)	T-score
L2	1.15	−0.5	92.1	−2.9
L3	1.00	−1.8	85.0	−3.2
L4	1.15	−0.5	85.5	−3.2
Average (L2-L4)	1.10	−1.0	87.6	−3.1

Abbreviations: BMD, bone mineral density; DXA, dual-energy x-ray absorptiometry; QCT, quantitative computed tomography; ROI, region of interest.

Case 1 demonstrates the limitations of DXA in assessing skeletal fragility in T2DM and the need for alternative imaging modalities that may provide additional insight into a patient's bone status. Although BMD measurements via different modalities cannot be directly compared and T-scores are not equivalent, the various imaging modalities were helpful in highlighting skeletal changes that were suggestive of poorer bone health that may have contributed to ongoing fractures while on antiresorptive therapy. Despite only a moderately reduced BMD by DXA, this patient’s TBS and QCT parameters were both low, which prompted a change in management for her fracture while on therapy.

## Measurement of Bone Parameters in Type 2 Diabetes

### Bone Mineral Density

DXA is the clinical standard for determining BMD and is strongly associated with fracture risk in the general population. Studies have shown that when matched for sex and age, people with T2DM have higher BMD compared with those without T2DM, particularly at the hip [38, 39]. This is predominantly driven by the higher BMI in patients with T2DM [40, 41]. Although BMD does predict fracture risk in T2DM, fracture risk is underestimated and for the same BMD, fracture risk in a T2DM person is equivalent to a non-T2DM counterpart with a T-score of 0.4 to 0.5 SD lower [42].

### Bone Microarchitecture

The hallmark of diabetic osteopathy is impaired microarchitecture. As exemplified in Case 1, DXA is unable to assess bone microarchitecture due to its low resolution. QCT uses three-dimensional reconstructions of bone that characterize bone structure, composition, and geometry. Higher spatial resolution means that individual trabeculae can be visualized within the bone, thereby allowing specific identification of bony trabecular and cortical compartments and providing a noninvasive assessment of bone microarchitecture. In contrast to an estimated areal BMD (aBMD; as is done by DXA), a true volumetric BMD (vBMD) can be calculated when the density is compared to a reference phantom of known density.

QCT is therefore a useful alternative to DXA when artifacts may affect accurate measurements, or when microarchitectural deficits are likely to be contributing. However, higher radiation precludes routine clinical use. High-resolution peripheral QCT (HR-pQCT) is limited to the distal radius and tibia and minimizes total body radiation, but it is currently limited to research use only and is not widely available.

### Quantitative Computed Tomography

There are few studies characterizing QCT in subjects with T2DM. As with areal BMD on DXA, spinal vBMD by QCT appears to be elevated in T2DM, although sex and ethnicity may mediate these changes [43, 44]. In a small cross-sectional study of postmenopausal women with and without T2DM and/or fracture, subjects with T2DM had higher vBMD and thicker cortical measurements at the hip [45]. However, in those with T2DM and fracture, these beneficial features were lost, and they had significantly thinner femoral cortices and larger femoral bone volume compared with those with T2DM alone. Causality cannot be ascertained in this cross-sectional study and the generalizability of these findings needs to be determined, but the observations do suggest that impaired cortical features may be associated with fracture risk.

### High-Resolution Peripheral Quantitative Computed Tomography

HR-pQCT allows for assessment of trabecular and cortical bone of the distal radius and tibia with minimal radiation. T2DM is associated with preserved trabecular bone but higher cortical porosity, particularly at the tibia [46, 47]. Studies attempting to identify the T2DM-related characteristics contributing to cortical porosity have shown a positive correlation between cortical deficits with microvascular complications [48] and peripheral vascular disease [49]. One study found higher fasting glucose was associated with unfavorable cortical bone microarchitecture, suggesting that hyperglycemia may be involved in the mechanism of skeletal fragility [50]; however, there was no correlation between HbA1c or T2DM duration and bone microarchitecture in another [48]. As with most studies assessing bone outcomes in T2DM, significant heterogeneity among subjects may confound results and it is not possible to establish a causal relationship between T2DM characteristics and HR-pQCT features in cross-sectional studies. Nevertheless, a recent meta-analysis confirmed adverse cortical characteristics on HR-pQCT in clinically heterogenous subjects with T2DM [51]. In particular, T2DM was associated with increased cortical porosity despite increased cortical thickness and preserved trabecular parameters. These adverse effects were more pronounced at the radius compared with the tibia suggesting that mechanical loading may mediate these changes. In contrast, subjects with type 1 diabetes mellitus had reduced trabecular vBMD and trabecular number, suggesting T2DM-specific factors (ie, obesity, insulin resistance) may play an important role in bone microarchitecture.

### Trabecular Bone Score

TBS quantifies gray-level pixelations of the lumbar spine to assess bone microarchitecture on a standard DXA scan. It can assist with determination of fracture risk, independent of BMD in the general population [52], and thus may be a useful tool for assessing microarchitecture particularly in patients with preserved BMD. T2DM is associated with reduced TBS on DXA, with suboptimal glycemic control resulting in worsening TBS and increased fracture risk [53-55]. However, TBS is affected by abdominal obesity, and this may be a confounder in T2DM subjects and may explain the observed differences in effects on trabecular bone compared to HR-pQCT.

### Bone Turnover

Normal bone remodeling is a tightly regulated, continuous process of coupled bone resorption orchestrated by osteocytes, osteoclasts, and osteoblasts. When this process is uncoupled, there is deterioration in the bone microarchitecture that leads to increased fracture risk, independent of BMD [56]. Tetracycline-labeled iliac bone biopsy histomorphometry provides direct assessment of bone turnover, but this is limited clinically due to its invasiveness. Serum bone turnover markers (BTMs) are a useful and practical surrogate that can be used in clinical practice.

Procollagen type 1 N-terminal propeptide (P1NP) and C-telopeptide (CTX) are serum BTMs that reflect bone formation and resorption respectively. Meta-analyses have suggested that bone turnover is lower in patients with T2DM, compared with counterparts who do not have T2DM [57-59]. The concept of hyperinsulinemia driving suppressed bone turnover in T2DM has been supported in a study using hyperinsulinemic-euglycemic clamps [60]. The authors found a negative correlation between BTMs and insulin resistance, with the lowest bone turnover identified in insulin-resistant and T2DM subjects independent of adiposity. Similarly, in a cross-sectional study, both T2DM and insulin resistance were associated with lower BTMs, independent of adiposity and body weight [41]. However, the role of BTMs in predicting fracture risk in T2DM remains unknown, with one case-control study finding no association in older subjects with T2DM [61].

In summary, T2DM is associated with relatively preserved BMD that is attributable to concomitant obesity. Despite higher BMD, fracture risk is increased, likely due to microarchitecture degradation, particularly cortical porosity, and low bone turnover. DXA is inadequate at assessing bone microarchitecture, thus alternative imaging modalities that can characterize microarchitecture may more accurately quantify the skeletal deficits in patients with T2DM and identify those at increased fracture risk.

## Calculating Fracture Risk in T2DM

Fracture risk calculators provide a personalized estimate of an individual's absolute risk of fracture to guide treatment thresholds. The Garvan Fracture Risk Calculator and Fracture Risk Algorithm (FRAX) are the most commonly used fracture calculators. However, they fail to take into consideration the unique impact of T2DM on bone fragility and consequently underestimate fracture risk, even when BMD is included. In light of this, T2DM has recently been added to the beta version of FRAXplus, an additional paid add-on to FRAX. The only freely available calculator that includes T2DM as a variable in risk fracture prevention is QFracture, but its use is limited outside of the UK. FRAX-determined fracture risk may more accurately reflect the true fracture risk in patients with T2DM by making any 1 of the 4 following adjustments: (i) adding 10 years of age; (ii) reducing BMD T-score by 0.5 SD; (iii) including rheumatoid arthritis as a substitute for T2DM; or (iv) by including TBS [62]. However, fracture risk remains underestimated even when any one of these adjustments is used. Despite underestimated fracture risk, fracture risk calculators and BMD measurements remain useful in assessing fracture risk in T2DM.

**Figure 2.**
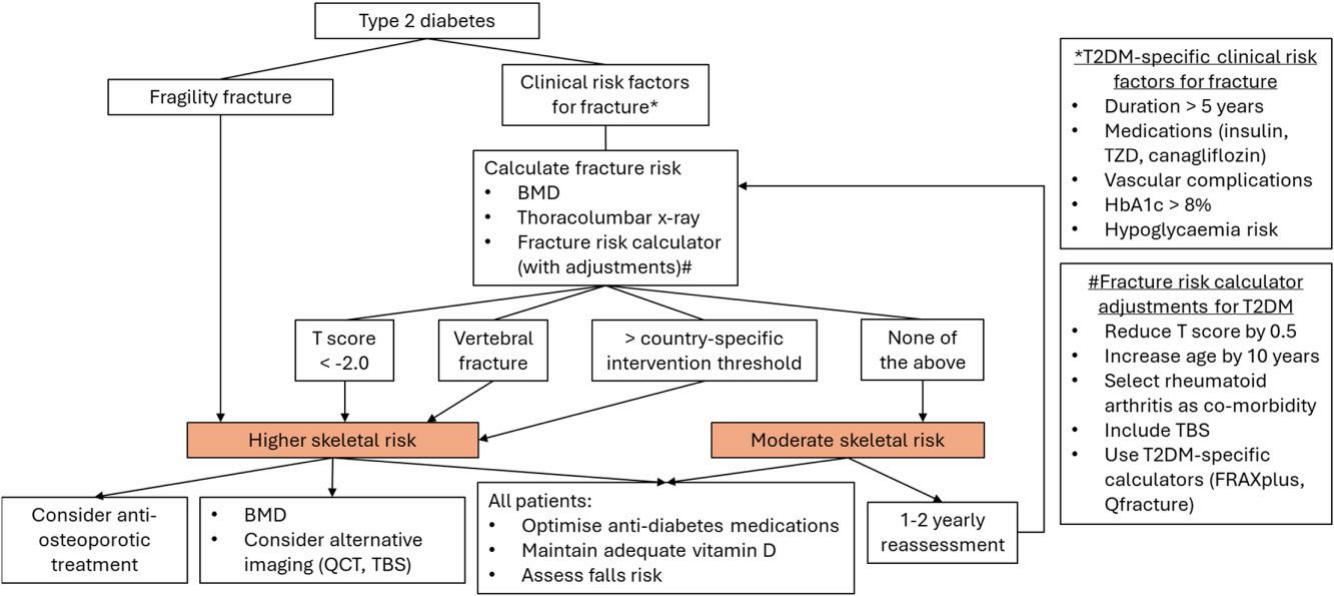
Managing skeletal risk in patients with T2DM. Postfracture mortality is elevated following all fractures in T2DM; thus, each fragility fracture should be a sentinel event that warrants specific management. As BMD may be relatively preserved, alternative imaging that can assess microarchitecture (eg, TBS, QCT) may be useful in quantifying skeletal deficits. In patients with fractures, BMD is useful for ongoing treatment monitoring. In patients without known fractures, further skeletal assessment should be performed in those with risk factors for fractures (including general clinical risk factors). Patients with low BMD, a vertebral fracture, or high risk based on a fracture risk calculator (with adjustments applied for T2DM) are at higher skeletal risk. All patients should have a review of their T2DM medications with preference for agents with neutral/positive bone effects (see Table 2). Abbreviations: BMD, bone mineral density; QCT, quantitative computed tomography; TBS, trabecular bone score; TZD, thiazolidinediones.

An algorithm for assessing and managing skeletal fragility in patients with T2DM is outlined in Fig. 2. There is a paucity of evidence-based data on the optimal management of these patients. Several groups, including the Bone and Diabetes Working Group of IOF [63], an interdisciplinary panel [64], and the American Diabetes Association [65] have developed guidelines for managing skeletal health in T2DM, which we have adapted and updated. Postfracture mortality is increased following fragility fractures at all sites, and therefore all fragility fractures represent higher skeletal risk that warrants consideration for therapy. Given that fracture risk is higher for the same BMD in T2DM, a T-score of −2.0 should be considered the threshold for significantly increased fracture risk in T2DM [62]. If available, QCT and/or TBS may reveal additional skeletal changes and could be particularly useful in patients in whom the severity of the clinical phenotype is discordant with BMD results (eg, in a patient with multiple fractures or recurrent fractures on therapy despite reasonable/improving BMD), although the lack of normative comparative data makes interpretation of the microarchitecture parameters difficult. Measuring BMD in a patient with established fragility fractures remains informative for monitoring treatment effect and duration.

## Case 2: Management of Skeletal Fragility in T2DM

A 58-year-old female was reviewed for osteoporosis. Her fracture history was significant for a surgical neck of humerus fracture after a fall from standing height. She had T2DM, managed with Novomix30® with a HbA1c of 7.2%. Her BMI was 29 kg/m^2^. She had no personal or family history of cardiovascular disease. Her DXA showed L1-L3 0.90 g/cm^2^, T-score −2.3 (significant degenerative changes) and left total femur 0.65 g/cm^2^, T-score −3.1. A thoracolumbar spine x-ray revealed an L2 vertebral compression fracture with > 20% height loss. She had received a single dose of 5 mg of intravenous zoledronic acid 12 months prior. She was commenced on romosozumab for management of skeletal fragility. For T2DM, she was prescribed semaglutide and metformin, with eventual insulin cessation.

Case 2 highlights the considerations when prescribing anti-osteoporotic and antidiabetic medications. Antidiabetic medications have varying effects on BMD and fracture risk. Insulin has been associated with increased fracture risk; however, the underlying mechanism remains unclear. Antidiabetic agents that minimize fracture risk should be considered in those at high skeletal risk. Considering that T2DM is a low bone turnover state, there is a theoretical advantage of anabolic bone therapy in this population, although there are no dedicated prospective trials examining this.

## Diabetes Medications and Their Effect on the Skeleton

Diabetes medications have variable effects on the skeleton (summarized in Table 2). There are no prospective trial data on the impact of antidiabetes agents on bone health, although observational studies and post hoc analyses provide important insights into the impact of these medications on fracture risk. Overall, agents that are neutral with respect to the skeleton should be preferentially used if possible. Agents that reduce risk of/progression of vascular complications (which appear to be independently associated with fracture risk) may have additional benefits in reducing fracture risk, although prospective trials have not been performed. Finally, careful consideration of hypoglycemia and falls risk is important in T2DM patients at high skeletal risk.

**Table 2.** Diabetes medications and their associated effects on BMD and fracture risk

Medication	Associated BMD	Associated fracture risk
Metformin	↔/↑ [66, 67]	↓/↔ any fracture [66, 67]
Sulphonylureas	↔ [68]	↑ nonvertebral fractures in men [69] ↔ vertebral fractures [70] ↓/↔ any fracture [66, 67]
Thiazolidinediones	↓ [71, 72]	↑ in women [71, 73]
DPP-4 inhibitors	↑ [74]	↓ [75, 76] ↑/↔ with saxagliptin [77, 78]
GLP1-RA	↔/↑ [79]	↓ [80] ↑/↔ with exenatide [81]
SGLT2-i	↑ with canagliflozin [82] ↔ with dapagliflozin [83]	↑ [84]/↔ [85] with canagliflozin ↔ [86-88]
Insulin	↑	↑ [9, 21]

Legend: ↔ no effect; ↑ increased; ↓ decreased.

Abbreviations: BMD, bone mineral density; DPP-IV inhibitors, dipeptidyl peptidase-4 inhibitors; GLP1-RA, glucagon-like peptide-1 receptor agonists; SGLT2-i, sodium glucose co-transport-2 inhibitors.

### Metformin

While there are no RCT data on metformin, large cohort studies have suggested a positive or neutral effect on BMD and fracture risk [66, 67].

### Sulfonylureas

The data on sulfonylurea use and bone health have yielded conflicting results, with an overall neutral or reduced fracture risk in some studies [66-68]. A multicenter observational study of diabetic elderly men found an increased risk in nonvertebral fractures, which may, in part, be related to hypoglycemia-induced falls [69].

### Thiazolidinediones

The impact of thiazolidinediones (TZDs) on bone metabolism and fracture risk has been investigated in both in vivo and in vitro studies, with an overall negative impact on bone health. Mechanisms include increased adipogenesis, impaired osteoblast differentiation and reduced bone formation [89]. An RCT of 50 postmenopausal women showed reduced bone formation and BMD after 14 weeks of treatment with rosiglitazone [72]. A meta-analysis of 10 RCTs and 2 observational studies showed a significantly increased risk of fractures in women treated with rosiglitazone or pioglitazone (OR 1.45 [95% CI, 1.18-1.79%]) [73]. A subsequent meta-analysis of 22 RCTs in both women and men confirmed the increased risk of fracture in women but no difference in men [71]. Fracture risk was independent of age, duration of use, and specific medication, suggesting a class effect.

### Dipeptidyl Peptidase-4 Inhibitors

Dipeptidyl peptidase-4 (DPP-4) inhibitors are associated with fracture risk reduction in a meta-analysis and large cohort study [75, 90]. One post hoc analysis evaluating the safety of saxagliptin in patients with T2DM found that the fracture incident rate was higher with saxagliptin (1.1 vs 0.6; incidence rates ratio 1.81 [95% CI 1.04-3.28)] [77]. However, there was no difference in risk of fracture in any of the large RCTs of saxagliptin and sitagliptin [78, 91] nor in a subsequent meta-analyses of multiple DPP-4 inhibitors [76].

### Glucagon-Like Peptide-1 Receptor Agonists

Weight loss has been associated with a decline in BMD; however, glucagon-like peptide-1 receptor agonists (GLP1-RAs) have been shown to successfully promote weight loss without increasing the risk of fractures. The use of exendin-4 on ovariectomized rats showed positive effects on biochemical markers of bone turnover (suggesting decreased bone resorption and increased bone formation). Exendin-4 treatment inhibited bone resorption and promoted bone formation, via the osteoprotegerin/RANKL pathways and direct osteoblastic stimulation, respectively [92]. A meta-analysis of RCTs of GLP1-RAs found a significantly reduced risk of incident fractures with liraglutide but an elevated risk with exenatide [81]. A subsequent larger meta-analysis, however, showed a reduction in fracture risk with all GLP1-RAs, including exenatide [80]. No studies have been powered for fracture outcomes, and differences in follow-up and active comparators in studies may explain the observed differences in findings.

### Sodium Glucose Co-Transport-2 Inhibitors

Sodium glucose co-transport-2 inhibitors (SGLT2-i) exert effects on bone health via the fibroblast growth factor 23/1,25-dihydroxyvitamin D/parathyroid hormone axis, resulting in altered calcium and phosphate homeostasis [93]. There is significant interest in skeletal outcomes with these agents. In the first RCT (CANVAS) and a pooled analysis of canagliflozin, there was an increased risk of fractures despite increased BMD [84]. However, the CREDENCE trial found no difference in fracture rates with canagliflozin [85]. Subsequent meta-analyses of canagliflozin, dapagliflozin, and empagliflozin also found no difference in fracture rates [86, 87]. A placebo-controlled RCT of 182 patients receiving dapagliflozin in addition to metformin showed no meaningful change in bone turnover or BMD after 102 weeks of therapy [83]. The reasons for the increased fracture risk in the CANVAS study alone are unclear, but based on these results, canagliflozin should be avoided in patients with or at high risk for fracture.

### Insulin

Insulin use has been associated with an overall increase in the risk of fractures [9, 21, 69]. Insulin is osteoanabolic, through direct activation of osteoblasts, sparking debate as to whether this increased fracture risk is a direct result of exogenous insulin on bone or whether there are indirect effects, such as hypoglycemia-induced falls or increased fall impact due to weight gain from insulin therapy [20]. As patients requiring insulin therapy often have clinical features that are also associated with increased fracture risk (such as microvascular complications and longer T2DM duration), it remains unclear whether insulin itself is associated with fracture risk or whether it is a surrogate marker for the severity of T2DM and its associated complications [9]. Nevertheless, the association of insulin therapy, independent of other T2DM-related clinical characteristics in the well-characterized cohort of the FIELD study [19] does suggest that insulin therapy itself may contribute to fracture risk, and dedicated studies including falls and hypoglycemia would be useful to understand the underlying pathophysiology.

## Anti-Osteoporotic Agents in T2DM

T2DM is associated with low bone turnover and relatively high or normal BMD, fueling a theoretical debate about the suitability of anti-osteoporotic medications in these patients. There are no dedicated prospective RCTs examining the safety and efficacy of osteoporosis treatments in T2DM. However, current evidence suggests that anti-osteoporotic agents exhibit at least comparable efficacy in those with T2DM compared to those without, and show no signal for increased adverse effects. Specific considerations regarding renal function (caution for bisphosphonates), anticipated duration of treatment (especially in light of near-normal/normal BMD), and cardiovascular safety may influence specific medication choice. Patients should also be monitored for adequate 25(OH)vitamin D levels (higher doses of vitamin D supplementation may be required in patients with T2DM due to concomitant obesity) [94] and advised targeted physical therapy to improve balance and muscle strength and reduce frailty and falls. There is no data on the efficacy of anti-osteoporotic medications in those with normal BMD nor on the effects on microarchitecture in T2DM. Given the high postfracture mortality in T2DM and the potential for mortality benefit with antiresorptive therapies [95], specific trials examining mortality benefit in T2DM patients should be confirmed.

### Antiresorptive Agents

In a post hoc analysis of the Fracture Intervention Trial (FIT), alendronate treatment in women with T2DM was associated with an increase in BMD at all sites, similar to the results of the main trial [96]. A cohort study of patients exposed to anti-osteoporotic drugs showed that T2DM status did not affect the fracture efficacy of bisphosphonates and raloxifene [97]. Despite having lower BTMs at baseline, a post hoc analysis of 3 phase III Japanese trials showed further lowering of BTMs and improvement in BMD in patients with T2DM treated with risedronate [98]. No studies have specifically assessed zoledronic acid in patients with T2DM. However, in a recent meta-analysis of 15 RCTs using antiresorptive agents (including 3 using risedronate and 2 using zoledronic acid), bisphosphonates in T2DM were associated with a reduction in vertebral, nonvertebral, and all fractures [59].

Similarly, denosumab has shown fracture efficacy in patients with T2DM, with a post hoc analysis from the FREEDOM and extension trials showing similar BMD gains and vertebral fracture efficacy in patients with and without T2DM [99]. However, denosumab (compared to placebo) in T2DM was associated with increased nonvertebral fractures, in contrast to the main study findings. The cause for this discrepancy is not clear but may relate to the unexpectedly low number of fractures in the placebo-treated subjects with T2DM. Interestingly, denosumab may have benefits on glycemia. A single dose of denosumab (60 mg) in postmenopausal women improved HbA1c and hepatic insulin sensitivity [100]. A further case-control study of patients with T2DM and prediabetes treated with denosumab compared to calcium/vitamin D or bisphosphonate, showed a significant improvement in HbA1c and BMI at 6 and 12 months [101]. Denosumab has been shown to cause *DPP-4* gene suppression and it has been hypothesized that metabolic effects of denosumab may be more apparent in those with pre-existing metabolic dysfunction. Longer-term effects on glycemia and metabolic status are unknown. Care should be given to the initiation of denosumab in patients in whom treatment cessation may be considered (eg, younger patients, relatively preserved BMD), as cessation can lead to rapid bone loss [102].

### Menopausal Therapies

Menopausal hormone therapy may be a safe option for women within 10 years of menopause, although overall risk of breast cancer and cardiovascular risk should be taken into account, given the baseline increased risk of these conditions in T2DM. Raloxifene has been associated with a reduction in vertebral, hip, and forearm fractures in women with T2DM, similar to those without T2DM [97, 103, 104]. There is no specific data on the use of tibolone in women with T2DM.

### Anabolic Agents

Anabolic agents have profound effects on BMD and fracture risk, particularly when used as first-line therapy. Considering the low bone turnover state of patients with T2DM, anabolic agents may be particularly beneficial in this population, with potentially additional gains in BMD and fracture reduction compared to those without T2DM. Data for teriparatide in T2DM is limited to a post hoc analysis of an observational study (DANCE), which showed similar improvement in BMD and reduction in vertebral fractures in subjects with T2DM compared with their non-T2DM counterparts [105]. A pooled analysis of this and 3 additional studies showed that teriparatide was associated with greater reductions in all fractures in subjects with T2DM compared to those without [106]. Abaloparatide resulted in equivalent BMD and TBS improvements in the ACTIVE trial [107]. Anabolic therapies in diabetic mice increase bone formation and correct cortical porosity, leading to improved bone mechanical properties [108], suggesting the underlying pathophysiology of diabetic osteopathy may be specifically targeted by these agents.

Currently, there are no available data on the efficacy or safety of romosozumab in T2DM. Further studies are warranted, given the potential increase in cardiovascular events identified in the alendronate vs romosozumab RCT (ARCH trial) [109].

## Conclusions

Skeletal fragility in T2DM is complex and managing these patients can be challenging. The clinical heterogeneity of T2DM is reflected in bone health, with various underlying pathophysiological drivers of hyperglycemia, insulin resistance, obesity, and vascular complications contributing to poorer skeletal health. Despite relatively preserved BMD in T2DM, microarchitecture is impaired, with predominantly cortical porosity, and low bone turnover. Alternative imaging modalities that can quantify microarchitectural changes (eg, TBS) may reveal skeletal deficits that predispose to fractures, particularly in patients with normal/near-normal DXA-determined BMD. Diabetes status should be included when calculating fracture risk; if T2DM cannot be specifically included (as in QFracture or FRAXplus), using one of 4 possible adjustments to FRAX (increase age by 10 years; lower T-score by 0.5; including rheumatoid arthritis; or including TBS measurement) improves fracture risk calculation. As well as traditional risk factors for fracture, there are T2DM-related clinical characteristics that are associated with increased risk of fracture, which should prompt treating clinicians to consider bone assessment, including screening vertebral radiography for unrecognized vertebral fractures.

There are no dedicated prospective trials determining optimal assessment and management of bone in T2DM. However, treatment thresholds in T2DM should be adjusted compared to the general population, including at higher BMD T-score levels. Currently available treatments appear to be effective and safe in T2DM. Anabolic therapy may be particularly advantageous but further data are needed before they can be specifically recommended over other agents. Finally, T2DM management should be individualized to minimize hypoglycemia and falls and to avoid agents that may have negative skeletal effects (eg, insulin therapy, sulfonylureas, thiazolidinediones, and canagliflozin).

## Data Availability

Data sharing is not applicable to this article as no datasets were generated or analyzed during the current study.

## Abbreviations

AGE: advanced glycation end product
BMD: bone mineral density
BMI: body mass index
BTM: bone turnover marker
CTX: C-terminal telopeptide
DPP-4: dipeptidyl peptidase-4
DXA: dual-energy x-ray absorptiometry
FIELD: Fenofibrate Intervention and Event Lowering in Diabetes
GLP1-RA: glucagon-like peptide-1 receptor agonist
HbA1c: glycated hemoglobin
HR-pQCT: high-resolution peripheral quantitative computed tomography
OR: odds ratio
QCT: quantitative computed tomography
P1NP: procollagen type I N-terminal propeptide
RCT: randomized controlled trial
RR: risk ratio
T2DM: type 2 diabetes mellitus
TBS: trabecular bone score
vBMD: volumetric bone mineral density
